# Left-right relationship-aware 3D volume classification method

**DOI:** 10.1007/s11548-025-03567-y

**Published:** 2026-01-28

**Authors:** Masahiro Oda, Yuichiro Hayashi, Yoshito Otake, Masahiro Hashimoto, Toshiaki Akashi, Shigeki Aoki, Kensaku Mori

**Affiliations:** 1 Information Technology Center, Nagoya University, Furo-cho, Chikusa-ku, Nagoya, Aichi 4648601 Japan; 2 Graduate School of Informatics, Nagoya University, Furo-cho, Chikusa-ku, Nagoya, Aichi 4648601 Japan; 3 Graduate School of Science and Technology, Nara Institute of Science and Technology, Takayama-cho, Ikoma, Nara 6300192 Japan; 4 Research Center for Medical Bigdata, National Institute of Informatics, Hitotsubashi, Chiyoda-ku, Tokyo 1018430 Japan; 5 Department of Radiology, Keio University School of Medicine, Shinanomachi, Shinjuku-ku, Tokyo 1608582 Japan; 6 Department of Radiology, Juntendo University, Hongo, Bunkyo-ku, Tokyo 1138431 Japan

**Keywords:** Left-right relationship, Classification, 3D volume

## Abstract

**Purpose:**

This paper proposes a left-right (LR) relationship-aware classification model for 3D volumetric images (3D volume). Bilateral symmetry (LR relationship) is an essential property of the human body that can be used to detect abnormalities and understand anatomical structures. Checking the difference and similarity between the left and right anatomical structures is very important in diagnosis. We propose an LR relationship-aware classification model of 3D volume.

**Methods:**

The proposed model employs an image feature extraction process from LR symmetric positions of human anatomy from 3D volume. Due to variations in body position and individual anatomical structure, small positional gaps among LR corresponding anatomical structures can be observed in medical images. We developed a multi-shift symmetric feature extraction module to accommodate such positional gaps.

**Results:**

The model was applied to 3D volume classification tasks of the lung and brain. From experimental results, the proposed model achieved superior performances both in the lung and brain classification tasks compared to the previous models. The result indicates that the proposed model has generalized performance in classifying anatomical structures with bilateral symmetric or semi-symmetric structures.

**Conclusion:**

We proposed the LR relationship-aware classification model of 3D volume. The proposed model effectively extracts image features from LR symmetric positions. The multi-shift symmetric feature extraction module was employed to accommodate small positional gaps among LR corresponding positions. The experimental results of 3D volume classification tasks of the lung and brain showed that the proposed method achieved superior performances compared to the previous models. Our code is available at https://github.com/modafone/lr3dvolumeclassification.

## Introduction

Integrating anatomical knowledge into medical image processing algorithms enables high-performance and data-efficient methods. Bilateral symmetry is an essential property of human anatomy and organisms; if a body is cut along one plane, the two halves have structures that almost mirror each other. This property is utilized in anatomical structure understanding and abnormality detection in image processing. Bilateral symmetry can be observed in the anatomical structures of the brain and musculoskeletal system. The lungs and kidneys have semi-bilateral symmetric structures. This symmetry or semi-symmetry is violated in the presence of lesions. Finding asymmetric structures in bilateral symmetric organs is a practical approach to diagnosis. Some diseases cause bilateral symmetric abnormal regions. COVID-19 is a disease that typically causes bilaterally distributed ground glass opacities (GGOs) in the left and right lungs. In radiological image reading, checking the difference and similarity between the left and right anatomical structures is very important in diagnosis.

Most previous medical image processing methods for computer-aided diagnosis (CAD) utilize convolutional neural networks (CNNs) or vision transformers (ViT). For 3D CT volumetric image (volume)-based COVID-19 CAD, many CNN [1–4] and ViT [5, 6] based methods are proposed. However, because CNN performs local image processing, the global distribution of GGOs across the left and right lungs is difficult to consider. While ViT can consider global image features, a large number of training data is necessary.

Bilateral symmetry-aware (more specifically, left-right (LR) relationship-aware) medical image processing methods were proposed for the brain and lung. For the brain and pelvic bone, LR relationships are utilized in segmentation and abnormality detection [7–10]. For the lung, pulmonary nodules and pneumonia detection methods utilize LR relationships [11, 12]. However, these previous methods utilize organ-specific knowledge in their process. The organ-by-organ development of the diagnosis assistance method is less effective and time-consuming. Developing a generalized LR relationship-aware approach for medical image processing is necessary to improve the accuracy of diagnosis assistance systems and reduce the load on system developments.

We propose a generalized LR relationship-aware classification model for 3D volume that exploits anatomical knowledge of organs or abnormal regions. The model employs a novel image feature extraction process from LR symmetric positions of human anatomy in a 3D volume. This process is called a symmetric feature extraction (SymFE) block, and it extracts meaningful information about abnormalities and organs. Small positional gaps among LR corresponding positions can be observed in medical images due to variations in body position and individual anatomical structure. We developed a multi-shift symmetric feature extraction (MiltiSymFE) module to accommodate such positional gaps. The SymFE blocks and MiltiSymFE modules are integrated into the proposed model.

This paper’s contributions are (1) proposing a novel LR relationship-aware classification model that effectively extracts image features considering the LR relationship of human anatomy or abnormal regions and (2) proposing the MultiSymFE module, which reduces the effect of positional gap among LR corresponding positions in image feature extraction. (3) The proposed method achieved superior performances in lung and brain disease classifications compared to previous methods, even with a limited number of training data.

## Method

### Left-right relationship-aware classification model

#### Overall model

**Fig. 1:**
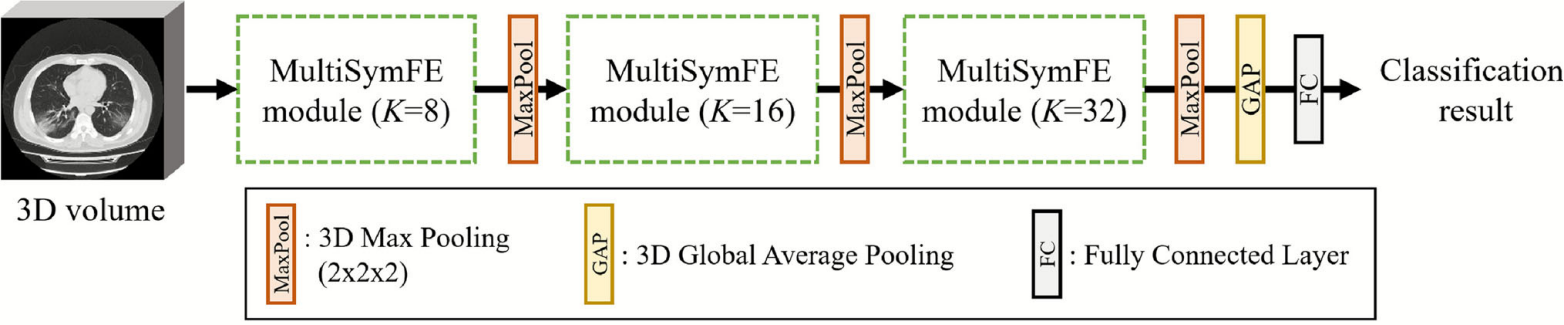
Overall architecture of left-right relationship-aware classification model. *K* is kernel number of 3D convolution operations used in modules

The proposed LR relationship-aware classification model performs the classification of a 3D volume. Its structure is shown in Fig. 1. It consists of MultiSymFE modules that perform image feature extraction considering positional gaps among LR corresponding anatomical structures. The MultiSymFE module performs image feature extractions using SymFE blocks with different positional gap settings among LR positions. The SymFE block performs feature extraction from LR symmetric positions in a volume using 3D convolution applied to LR symmetrical positions.

**Fig. 2:**
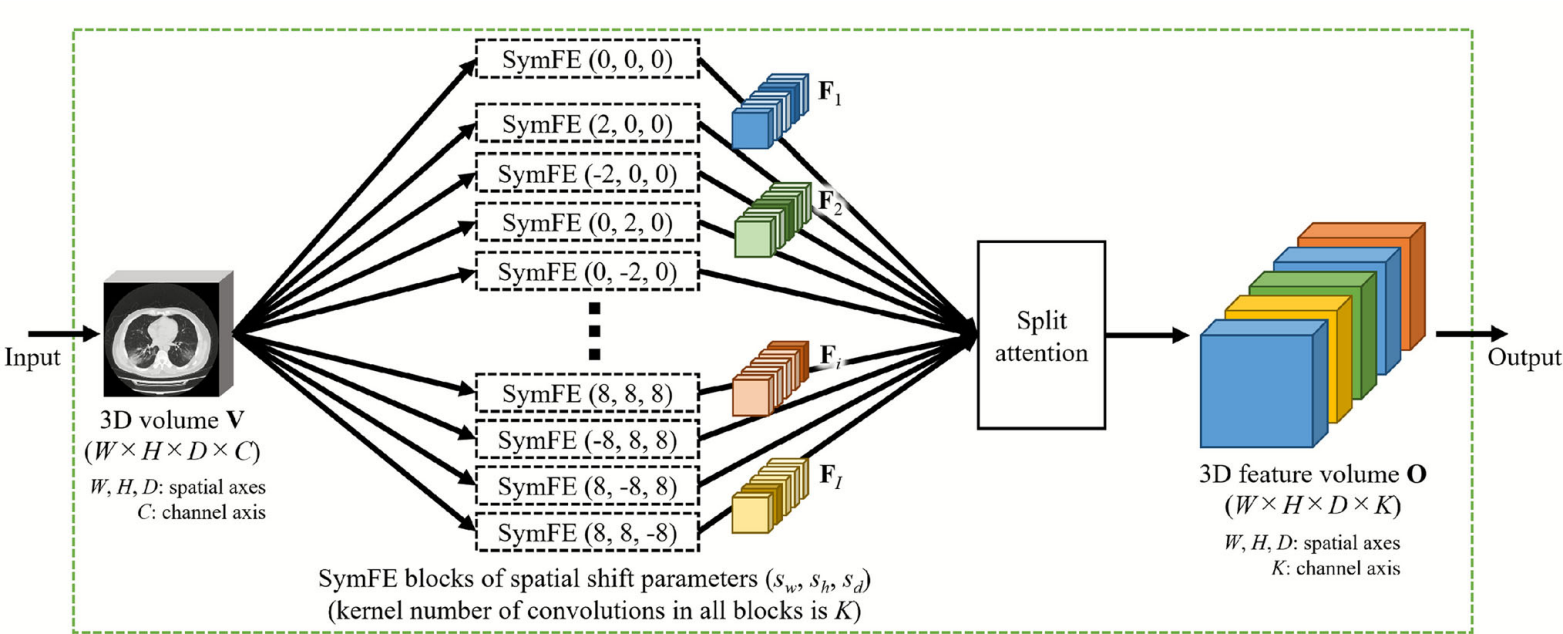
Architecture of MultiSymFE module. SymFE blocks having various spatial shift parameters are applied in parallel. 3D feature volumes obtained from them are selectively fused by split attention

#### Multi-shift symmetric feature extraction (MultiSymFE) module

This module extracts LR relationship-aware features considering positional gaps among LR positions from a 3D volume. Image features extracted from LR symmetry locations in a volume contain meaningful information about abnormalities and organs. However, small positional gaps among LR symmetry locations can be observed due to the variations among body positions and individual anatomical structures. The MultiSymFE module accommodates such positional gaps by integrating 3D feature volumes obtained by the SymFE blocks of various positional gap settings. The positional gap settings include multiple distances and directions in a volume. Figure 2 shows the module’s structure.

The input of the MultiSymFE module is a 3D volume $\textbf{V} \in {\mathbb {R}}^{W \times H \times D \times C}$ where *W*, *H*, *D* represent the volume size along the *x*, *y*, *z* axes and *C* represents the number of channels. $\textbf{V}$ is processed by multiple SymFE blocks that are connected in parallel. The kernel number of 3D convolutions applied in all SymFE blocks is *K*. Each SymFE block uses spatial shift parameters different from those of other blocks. The spatial shift parameter corresponds to the positional gap among LR symmetry locations in the 3D volume. We represent the spatial shift parameter as $(s_w, s_h, s_d)$, where each value means the difference of LR symmetry locations along the *x*, *y*, *z* axes of the 3D volume. We obtain many 3D feature volumes $\textbf{F}_{i} \in {\mathbb {R}}^{W \times H \times D \times K}$ from the SymFE blocks. Where $i \ (i=1,\ldots ,I)$ is the index of 3D feature volumes, and *I* is the total number of SymFE blocks.

We exploit the split attention [13] to selectively fuse $\textbf{F}_{i} \ (i=1,\ldots ,I)$ into an output 3D feature volume $\textbf{O} \in {\mathbb {R}}^{W \times H \times D \times K}$. The split attention performs feature volume attention across multiple 3D feature volumes. Split attention is calculated as follows. Global importance information for each channel in $\textbf{F}_{i}$ is given by global average pooling across spatial axes. Which is calculated by

1 $$\begin{aligned} s_{i,c} = \frac{1}{WHD}\sum ^{W}_{x=1} \sum ^{H}_{y=1} \sum ^{D}_{z=1} f_{i}(x,y,z,c), \end{aligned}$$

where $f_{i}(x,y,z,c)$ is an element of $\textbf{F}_{i}$ at index (*x*, *y*, *z*, *c*) and $c \ (c=1,\ldots ,K)$ is the index of channel. A weighted fusion of 3D future volumes is obtained using channel-wise attention, where each channel in the output $\textbf{O}$ is obtained as a weighted combination over $\textbf{F}_{i}$. The *c*-th channel in $\textbf{O}$ is given by

2 $$\begin{aligned} \textbf{O}_{c} = \sum ^{I}_{i=1} a_{i,c} \textbf{F}_{i,c}, \end{aligned}$$

where $\textbf{F}_{i,c}$ is the *c*-th channel in $\textbf{F}_{i}$. $a_{i,c}$ is an assignment weight given by

3 $$\begin{aligned} a_{i,c} = \frac{1}{1+e^{-s_{i,c}}}. \end{aligned}$$

From the split attention, we generate $\textbf{O}$ as the output of this module.

#### Symmetric feature extraction (SymFE) block

**Fig. 3:**
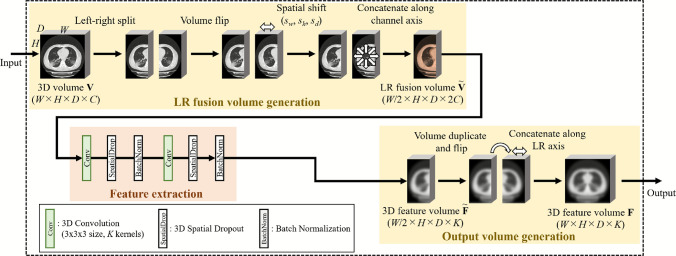
Architecture of SymFE block. This block extracts LR relationship-aware features from LR symmetric positions. $(s_w, s_h, s_d)$ is spatial shift parameter and *K* is kernel number of 3D convolution operations

This block extracts LR relationship-aware features from LR symmetric positions in a 3D volume. The input of this block is a 3D volume $\textbf{V} \in {\mathbb {R}}^{W \times H \times D \times C}$. Three processes, including the LR fusion volume generation, the feature extraction, and the output volume generation, are applied to $\textbf{V}$ as shown in Fig. 3.

We first apply the LR fusion volume generation process to $\textbf{V}$. In this process, $\textbf{V}$ is split along the left-right axis of the human body into two volumes of equal size. Voxel values in one of the split volumes are flipped along the left-right axis. Then, we apply a spatial shift operation of parameters $(s_w, s_h, s_d)$ to voxel values in the flipped volume. The two volumes, one of which is not changed after splitting and the other processed by the flip and shift operations, are concatenated along the channel axis. We call the concatenated volume as an *LR fusion volume* $\tilde{\textbf{V}} \in {\mathbb {R}}^{W/2 \times H \times D \times 2C}$. In $\tilde{\textbf{V}}$, voxel values at LR symmetry locations in the original volume $\textbf{V}$ are arranged at the same spatial location.

We apply the feature extraction process to $\tilde{\textbf{V}}$. We can extract feature values from LR symmetry locations in the original volume $\textbf{V}$ by applying 3D convolutions to $\tilde{\textbf{V}}$. We apply 3D convolutions of kernel size $3 \times 3 \times 3$ with *K* kernels to $\tilde{\textbf{V}}$. To reduce the overfitting of the model to training data, we apply 3D spatial dropout [14] (dropout probability is *p*) after the 3D convolutions. 3D feature volume ${\tilde{\textbf{F}}} \in {\mathbb {R}}^{W/2 \times H \times D \times K}$ is obtained from this process.

Finally, we apply the output volume generation process to ${\tilde{\textbf{F}}}$. ${\tilde{\textbf{F}}}$ is duplicated, and the duplicated volume is flipped along the left-right axis. Then, the flipped volume is combined (concatenated along the left-right axis) with ${\tilde{\textbf{F}}}$ to make a 3D feature volume $\textbf{F} \in {\mathbb {R}}^{W \times H \times D \times K}$ that has the same spatial size as $\textbf{V}$. $\textbf{F}$ is the output of this block.

### 3D volume classification method

#### Classification tasks

We apply the proposed model to two tasks, including *lung classification* and *brain classification*. In the *lung classification*, the model classifies a chest CT volume into two classes corresponding to a high or low likelihood of a COVID-19 case. In the *brain classification*, the model classifies a T1 contrast-enhanced (T1CE) MR volume into two classes that correspond to high-grade glioma (HGG) or low-grade glioma (LGG) cases.

#### Preprocessing

In the *lung classification*, we perform CT value normalization, which normalizes CT values within the range of $-2050$ H.U. to 950 H.U. to values from $-1.0$ to 1.0. CT values lower than $-2050$ H.U. and higher than 950 H.U. are replaced with $-1.0$ and 1.0, respectively. We apply the lung normal and infection region segmentation method [15] to the normalized volume. Segmented lung normal and infection regions are combined to make lung regions. We make a volume by removing axial slices that do not include the lung region from the normalized volume. The volume is scaled to $144 \times 144 \times 48$ voxels.

In the *brain classification*, we perform intensity value normalization of T1CE volume. Intensity values of volumes were normalized by scaling intensities to [0.5, 99.5] percentile, as performed in the preprocessing of the nnU-Net [16]. The normalized volume is scaled to $144 \times 144 \times 48$ voxels. In this task, segmentation or clipping of brain regions is unnecessary because we use a dataset containing skull-stripped T1CE volumes (dataset information is provided in Sect. 3).

#### Classification

The volume is classified into two classes using the proposed model. In the MultiSymFE module, we use 33 SymFE blocks having spatial shift parameters $(s_w, s_h, s_d) \in \{(0,0,0), (\pm 2, 0, 0)$, $(\pm 4, 0, 0)$, $(\pm 6, 0, 0)$, $(\pm 8, 0, 0)$, $(0, \pm 2, 0)$, $(0, \pm 4, 0)$, $(0, \pm 6, 0)$, $(0, \pm 8, 0)$, $(\pm 2, \pm 2, 0)$, $(\pm 4, \pm 4, 0)$, $(\pm 6, \pm 6, 0)$, $(\pm 8, \pm 8, 0)$}. (Note that $(\pm x, 0, 0)$ and $(\pm x, \pm y, 0)$ include two and four parameter settings, respectively.) Spatial shift along the *z* axis is set to 0 because the volume is small on the *z* axis.

## Experiments and results

We evaluated the classification performance of the proposed model in the two classification tasks explained in Sect. 2.2.

In the *lung classification*, we used 1507 CT volumes of our private dataset, including COVID-19 and non-COVID-19 cases. The CT volumes were obtained from multiple medical institutions in Japan. The ethical review committees in related institutions approved using our dataset in this research. Experienced radiologists annotated the ground truth class labels of CT volumes, including the high or low likelihood of COVID-19 (two classes). The radiologists evaluated CT volumes based on the expert consensus in COVID-19 diagnosis reported by the Radiological Society of North America (RSNA) [17].

In the *brain classification*, we used 285 T1CE MR volumes, including HGG and LGG cases provided by the Brain Tumor Segmentation (BraTS) 2018 dataset [18–20].

For both tasks, we conducted four-fold cross-validation in our evaluations. We evaluated the classification results using accuracy, F1 score, precision, and recall. Dropout probability was set as $p=0.1$. In training the models, we set the minibatch size as 8. All models were trained from scratch. We used the Adam optimizer and set the linear learning rate warmup to 0.005 in the initial 10 epochs, and cosine learning rate decay was used. Models were implemented using TensorFlow 2.4.0. One NVIDIA RTX A6000 GPU was used to train and test the methods.

### Lung classification: comparative study

We compared the classification performances of the proposed model with previously proposed classification models in the *lung classification* task. 3D CT volume classification models for COVID-19 or lung CAD proposed by Xue et al. [1] and Zunair et al. [21] were evaluated for comparison. We also compared the proposed model with widely used models. ResNet-101/152 [22], DenseNet-121/169/201 [23], EfficientNet-B0/B1/B2 [24], MobileNet [25], and MobileNetV2 [26] were proposed as 2D image classification models. We modified them to make 3D volume classification models. 3D ResNet-101/152, 3D DenseNet-121/169/201, 3D EfficientNet-B0/B1/B2, 3D MobileNet, and 3D MobileNetV2 were used in comparisons. Training epochs of all models were set as 80. The mean and standard deviation (S.D.) of classification performances are shown in Table 1. From this table, the proposed method achieved the highest performances in accuracy, F1 score, and precision. We performed the two-sided t-test between the proposed model and each previous model to evaluate statistically significant (*p*-value < 0.05) performance improvements. The results are also shown in Table 1.

**Table 1:** Classification performances of proposed and previously proposed models in *lung classification* task

Model	Accuracy (%)	F1 score (%)	Precision (%)	Recall (%)
Proposed	**81**.**5** ± 1.73	**84**.**6** ± 1.51	**86**.**9** ± 1.56	82.3 ± 1.67
Xue et al. [1]	73.1 ± 1.48*	78.5 ± 1.37*	77.5 ± 3.05*	79.5 ± 1.01*
Zunair et al. [21]	79.3 ± 1.62	82.5 ± 1.40	86.3 ± 0.60	79.1 ± 2.77
3D ResNet-101	73.3 ± 1.86*	78.6 ± 1.87 *	77.6 ± 2.15*	79.7 ± 2.61
3D ResNet-152	73.1 ± 1.59*	78.3 ± 1.24*	78.1 ± 3.01*	78.5 ± 1.65*
3D DenseNet-121	75.5 ± 0.67*	80.6 ± 0.71*	78.7 ± 1.37*	**82**.**7** ± 2.08
3D DenseNet-169	75.2 ± 1.98 *	80.1 ± 1.66 *	79.4 ± 0.75 *	80.9 ± 2.90
3D DenseNet-201	73.7 ± 1.75 *	79.1 ± 1.24 *	77.9 ± 1.79 *	80.4 ± 2.61
3D EfficientNet-B0	65.4 ± 2.17 *	71.6 ± 2.12 *	72.7 ± 4.02 *	70.7 ± 3.17 *
3D EfficientNet-B1	77.5 ± 2.00 *	81.6 ± 1.08 *	82.5 ± 2.80	80.9 ± 1.44
3D EfficientNet-B2	78.9 ± 1.52	82.5 ± 0.80	84.5 ± 2.21	80.7 ± 2.60
3D MobileNet	75.4 ± 2.90 *	80.0 ± 2.42 *	80.4 ± 2.11 *	79.6 ± 3.29
3D MobileNetV2	76.6 ± 3.30	81.0 ± 2.78	81.3 ± 2.61 *	80.8 ± 4.07

Mean and SD among four-fold cross-validation are listed. Bold values indicate best performances

*Indicates statistical significance (*p*-value < 0.05) between proposed and each previous model

### Brain classification: comparative study

We compared the classification performances of the proposed model with previously proposed classification models in the *brain classification* task. Training epochs of all models were set as 60. The mean and S.D. of classification performances are shown in Table 2. The proposed method achieved the highest performances in accuracy, F1 score, and precision. We performed the two-sided t-test between the proposed model and each previous model to evaluate statistically significant (*p*-value < 0.05) performance improvements. However, no significant difference was observed between the proposed model and previous models.

**Table 2:** Classification performances of proposed and previously proposed models in *brain classification* task

Model	Accuracy (%)	F1 score (%)	Precision (%)	Recall (%)
**Proposed**	**78**.**1** ± 2.70	**87**.**0** ± 1.72	**77**.**4** ± 2.67	99.5 ± 0.82
3D ResNet-152	74.8 ± 1.20	85.2 ± 1.09	75.2 ± 1.12	98.1 ± 1.25
3D DenseNet-121	75.6 ± 2.03	85.7 ± 1.24	75.4 ± 2.23	99.5 ± 0.85
3D DenseNet-169	75.5 ± 1.18	85.7 ± 0.86	75.3 ± 1.55	99.5 ± 0.85
3D DenseNet-201	74.9 ± 2.63	85.4 ± 1.56	75.1 ± 2.59	99.0 ± 0.96
3D EfficientNet-B0	76.1 ± 0.87	86.0 ± 0.48	75.8 ± 0.98	99.5 ± 0.85
3D EfficientNet-B1	74.2 ± 2.33	85.1 ± 1.51	74.3 ± 2.39	99.5 ± 0.85
3D EfficientNet-B2	75.9 ± 1.65	85.9 ± 1.01	75.6 ± 1.91	99.5 ± 0.85

Mean and SD among four-fold cross-validation are listed. Bold values indicate best performances

### Ablation study

The MultiSymFE module extracts image features by considering multiple positional gaps among LR positions within a 3D volume. Consideration of multiple positional gaps in the module is performed through the parallel process of SymFE blocks having various spatial shift parameters as shown in Fig. 2. In this module, the use of multiple SymFE blocks with different spatial shift parameters ($s_w, s_h, s_d$) contributes to improving the classification performance of the proposed model. We confirmed the relationship between the number of SymFE blocks and the classification performances of the proposed model in an ablation study.

We proposed models with different numbers of SymFE blocks in the MultiSymFE module: 33, 25, 17, 9, and 1. Spatial shift parameter settings in the SymFE blocks of the models are shown in Table 3.

We evaluated the classification performances of the proposed models with different numbers of SymFE blocks. The results are shown in Table 4. From the results, the use of many SymFE blocks contributed to performance improvement.

**Table 3:** Spatial shift parameter settings of proposed models used in ablation study

Num. of SymFE blocks	Spatial shift parameters
33 SymFE blocks	$$\begin{array}{l}{(s_w, s_h, s_d)_{33} \in \{(0,0,0), (\pm 2, 0, 0), (\pm 4, 0, 0), (\pm 6, 0, 0), (\pm 8, 0, 0)}, \\ {(0, \pm 2, 0), (0, \pm 4, 0), (0, \pm 6, 0), (0, \pm 8, 0), (\pm 2, \pm 2, 0), (\pm 4, \pm 4, 0)}, \\ {(\pm 6, \pm 6, 0), (\pm 8, \pm 8, 0) \}}\end{array}$$
25 SymFE blocks	$$\begin{array}{l} {(s_w, s_h, s_d)_{25} \in \{(0,0,0), (\pm 2, 0, 0), (\pm 4, 0, 0), (\pm 6, 0, 0)}, \\ {(0, \pm 2, 0), (0, \pm 4, 0), (0, \pm 6, 0), (\pm 2, \pm 2, 0), (\pm 4, \pm 4, 0), (\pm 6, \pm 6, 0) \} }\end{array}$$
17 SymFE blocks	$$\begin{array}{l} {(s_w, s_h, s_d)_{17} \in \{(0,0,0), (\pm 2, 0, 0), (\pm 4, 0, 0)}, \\ {(0, \pm 2, 0), (0, \pm 4, 0), (\pm 2, \pm 2, 0), (\pm 4, \pm 4, 0) \} }\end{array}$$
9 SymFE blocks	$$\begin{array}{l} {(s_w, s_h, s_d)_{9} \in \{(0,0,0), (\pm 2, 0, 0), (0, \pm 2, 0), (\pm 2, \pm 2, 0) \} } \end{array}$$
1 SymFE block	${(s_w, s_h, s_d)_{1} = (0,0,0)}$

Note that $(\pm x, 0, 0)$ and $(\pm x, \pm y, 0)$ include two and four parameter settings, respectively

**Fig. 4:**
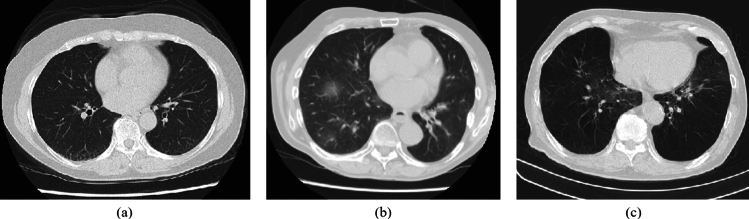
COVID-19 cases that were correctly classified by proposed model, having 33 SymFE blocks, but misclassified by proposed model, having 1 SymFE block

Figure 4 shows axial slice images of COVID-19 cases correctly classified by the proposed model with 33 SymFE blocks but misclassified by the proposed model with 1 SymFE block. In these cases, the patients’ body positions were slightly rotated from the ideal supine position. The use of multiple SymFE blocks with different spatial shift patterns in the MultiSymFE module enabled the correct classification of cases with variations in body positions.

**Table 4:** Classification performances in ablation study using different numbers of SymFE blocks

Model	Accuracy (%)	F1 score (%)	Precision (%)	Recall (%)
Model w/33 SymFE blocks	**81**.**5** ± 1.73	**84**.**6** ± 1.51	**86**.**9** ± 1.56	**82**.**3** ± 1.67
Model w/25 SymFE blocks	79.5 ± 1.16	82.6 ± 1.62	86.3 ± 0.67	79.3 ± 2.97
Model w/17 SymFE blocks	80.1 ± 1.36	83.3 ± 1.42	86.6 ± 2.01	80.2 ± 2.06
Model w/9 SymFE blocks	79.3 ± 1.64	82.9 ± 1.76	86.3 ± 0.35	79.8 ± 3.28
Model w/1 SymFE block	74.1 ± 4.49	77.7 ± 7.55	82.9 ± 10.16	77.9 ± 17.9

Mean and SD among four-fold cross-validation are listed. Bold values indicate best performances

**Fig. 5:**
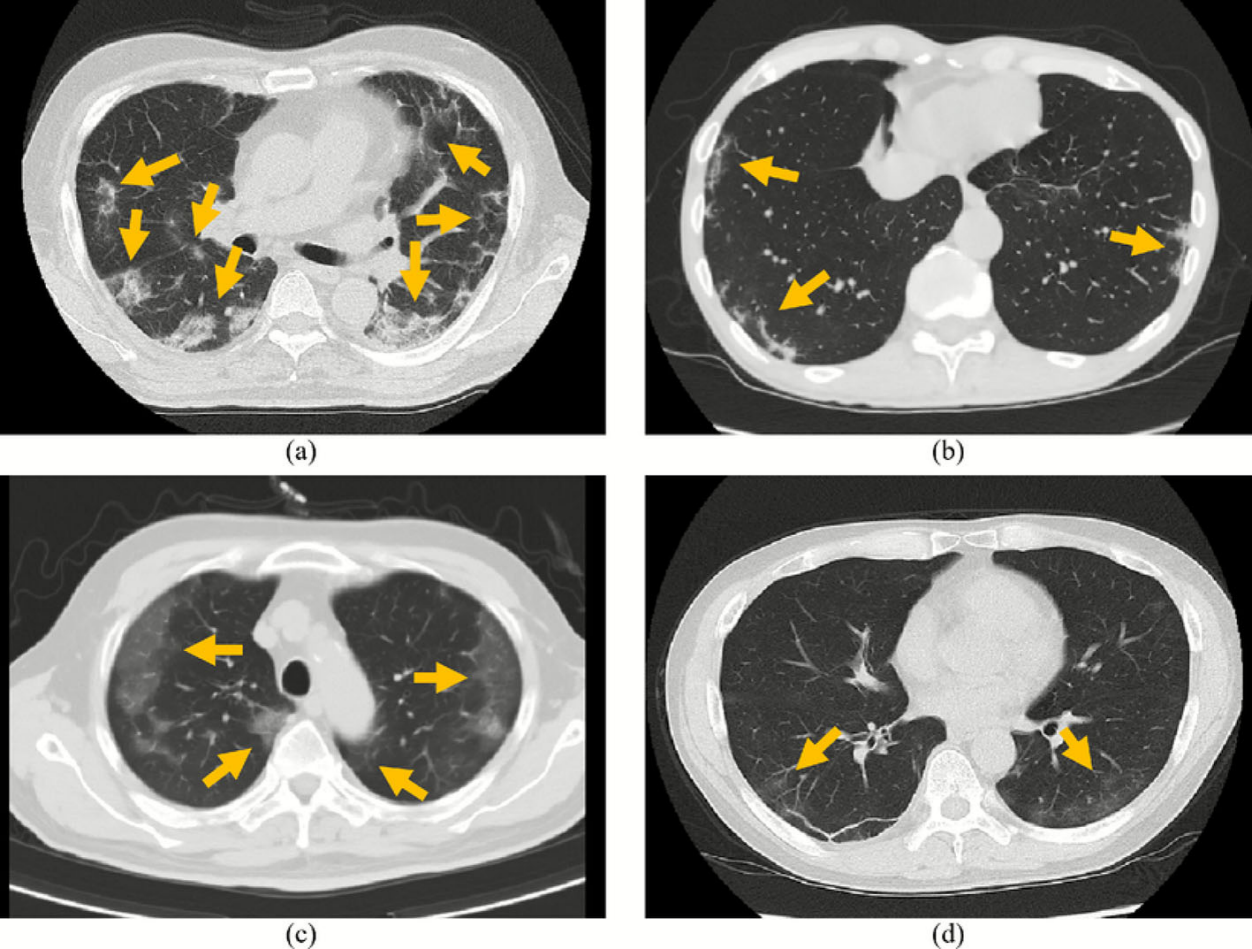
TP cases of classification result from proposed model. GGOs are indicated by arrows

**Fig. 6:**
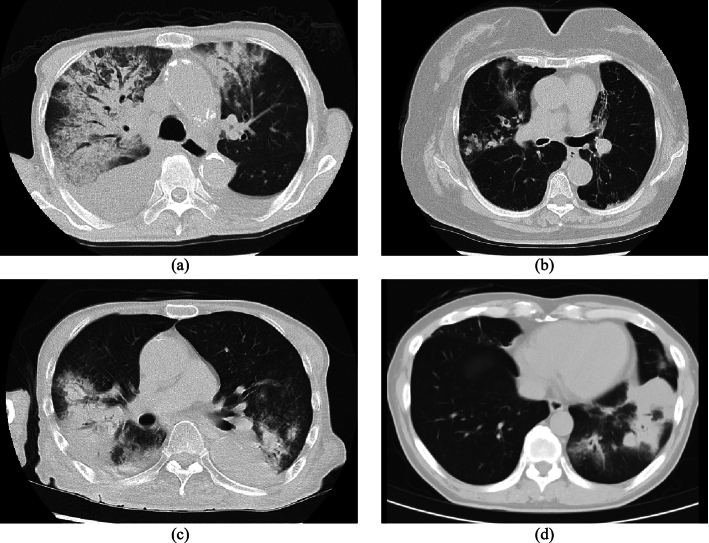
FP cases classified by the proposed model. Many abnormal regions, including GGOs and consolidations, were found that were caused by disorders other than COVID-19

**Fig. 7:**
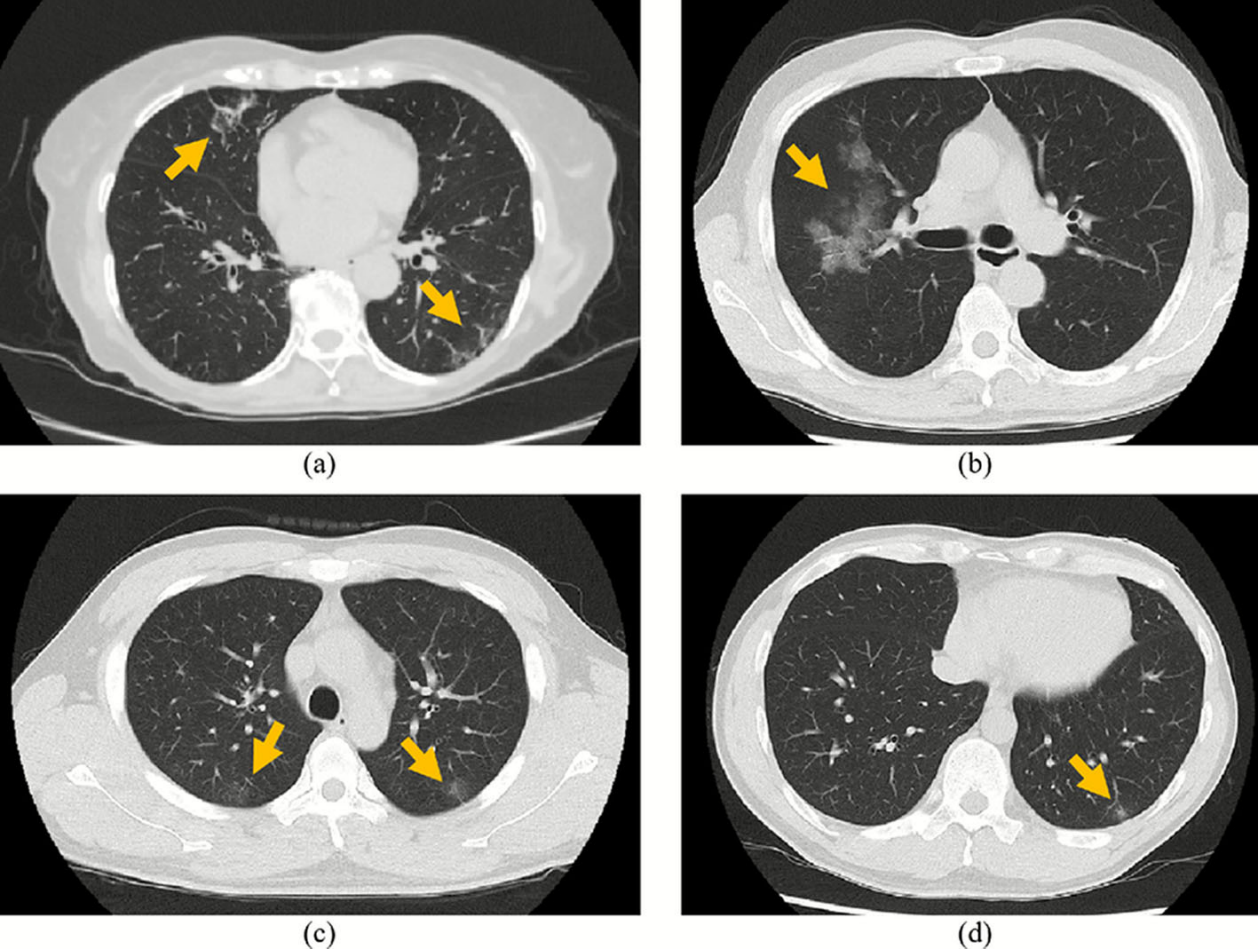
FN cases classified by the proposed model. GGOs are indicated by arrows

### Qualitative results

We qualitatively evaluated axial slice images of the classification results of the proposed model in the *lung classification task*.

Figure 5 shows true-positive (TP) cases. Many GGOs were found in the lung regions caused by COVID-19 infection. They were semi-bilaterally distributed in the left and right lungs. GGOs showed variations in their intensity and shape. For example, GGOs in Fig. 5a, c had high intensities, whereas those in Fig. 5b, d had low intensities. Furthermore, GGOs in Fig. 5b, d were very small. The proposed model correctly classified these cases as having a high likelihood of COVID-19.

Figure 6 shows false-positive (FP) cases. We found many abnormal regions, including GGOs and consolidations in the lung, that were caused by disorders other than COVID-19. In such cases, the distribution of GGOs and consolidations was similar to that of COVID-19 cases. It makes classification difficult.

Figure 7 shows false-negative (FN) cases. GGOs were found in FN cases. However, as shown in Fig. 7, GGOs in FN cases were small and had very low intensities. They are difficult to find, even for humans.

## Discussion

In both comparative studies, the proposed model achieved the highest classification performance in terms of accuracy, F1 score, and precision. Furthermore, the proposed model showed significantly higher performances than many previously proposed models in the lung classification task. The results indicate that the proposed model has superior and generalized performance in processing anatomical structures with bilateral symmetry or semi-symmetry. While CNNs are effective for local image feature extraction, global image features are underutilized. ViT has the potential to perform global image feature-based processing. Global image features are achieved by the multi-head self-attention (MHSA) mechanism used in ViT. However, a large amount of training data is required to achieve satisfactory results with MHSA. Correcting a large amount of data is difficult in medical image processing. Our proposed model was designed based on anatomical knowledge. Under the assumption that structures having LR relationships are included in the 3D volume, the proposed model can extract and utilize local and global image features for classification. Furthermore, the proposed model can be trained with a reasonable number of training data to achieve high classification performance. The proposed model is quite helpful for clinical image analysis.

In the ablation study, using many SymFE blocks improved classification performances. This result indicates that considering positional gaps between LR symmetric locations is essential. We utilized 33 spatial shift patterns (described in Sect. 2.2) in the MultiSymFE module. These patterns include multiple settings of distance and directions of positional gaps. The use of multiple patterns contributed to the correct classification of cases with variations in patient body position, as shown in Fig. 4. Using more shift patterns, including shifts along the *z*-axis, will improve classification performance. However, the amount of GPU memory limits the number of shift patterns. In our experiments, using 33 shift patterns consumed about 45 GB of GPU memory during training. To increase the number of shift patterns, either a single GPU with large memory or parallel processing across multiple GPUs will be necessary. Recently, some GPUs with large memories have been released and are commercially available. The number of spatial shift patterns in the MultiSymFE module could be improved by utilizing recently released GPUs, thereby enhancing the model’s performance.

In the qualitative evaluation, we analyzed the visual characteristics of axial slices from TP, FP, and FN cases. COVID-19 causes bilaterally distributed GGOs in both lungs. Cases having such characteristics were correctly classified as shown in Fig. 5. In FP cases shown in Fig. 6, non-COVID-19 cases having similar appearances to COVID-19 were misclassified. Intensities of GGOs and consolidations in FP cases tend to be higher than those of COVID-19 cases. Such differences in intensities can be utilized to improve the classification accuracy of the proposed model. In FN cases shown in Fig. 7, very small GGOs were observed. In preprocessing for the proposed model, we scaled a CT volume to $144 \times 144 \times 48$ voxels. Small GGOs are challenging to detect at scale. Multi-scale processing of the CT volume will be necessary to consider small objects in the classification process.

## Conclusions

In this paper, we proposed the LR relationship-aware classification model. The model extracts image features from LR symmetric positions in a 3D volume. Furthermore, the model accommodates small positional gaps among LR corresponding positions by fusing multiple patterns of volume shifting. We applied the proposed model to 3D volume classification tasks of the lung and brain. The application of the proposed model resulted in better classification performance than the previous models. The proposed model achieved the highest classification performances in accuracy, F1 score, and precision in the lung and brain volume classifications. Future work includes applying the model to other organs and introducing more anatomical knowledge into the model.
